# Shh–S100A10 axis curbs neuronal PANoptosis after ischemic stroke

**DOI:** 10.4103/NRR.NRR-D-24-01661

**Published:** 2025-09-29

**Authors:** Ling Wang, Hao Tang, Jun Wen, Qinghuan Yang, Jiagui Huang, Yong Zhao, Yu Ren, Qin Yang

**Affiliations:** 1 Department of Neurology, The First Affiliated Hospital of Chongqing Medical University, Chongqing, China; 2 Department of Neurology, the Second People’s Hospital of Yibin, Yibin, Sichuan Province, China

**Keywords:** acell death, cerebral ischemia, nerve regeneration, neurological function, neuron, PANoptosis, S100 calcium-binding protein A10, Sonic Hedgehog

## Abstract

Acute ischemic stroke is a highly prevalent and disabling disease with poor prognosis. Neuronal death is a major feature after a stroke. PANoptosis is a newly reported pattern of cell death, characterized by pyroptosis, apoptosis, and necroptosis, that plays an important role in the pathophysiological process after ischemic brain injury. However, its precise underlying mechanisms have not yet been fully elucidated. This study aimed to clarify the function of S100 calcium-binding protein A10 in neuronal PANoptosis after ischemic brain damage and to investigate the impact and mechanism of sonic hedgehog and S100 calcium-binding protein A10 on PANoptosis. The results showed that S100 calcium-binding protein A10 was significantly upregulated in both cellular and animal models of ischemic stroke. Knockdown of S100 calcium-binding protein A10 exacerbated PANoptosis and the levels of PANoptosis-related proteins following cerebral ischemia damage. Sonic hedgehog treatment increased S100 calcium-binding protein A10 and inhibited the increase in PANoptosis induced by S100 calcium-binding protein A10 knockdown. The findings suggest that sonic hedgehog intervention mitigated neuronal PANoptosis ensuing from ischemic stroke. The combination of S100 calcium-binding protein A10 and sonic hedgehog demonstrated promise for developing an effective therapy against cerebral ischemic stroke, which would have significant potential for future clinical applications.

## Graphical abstract

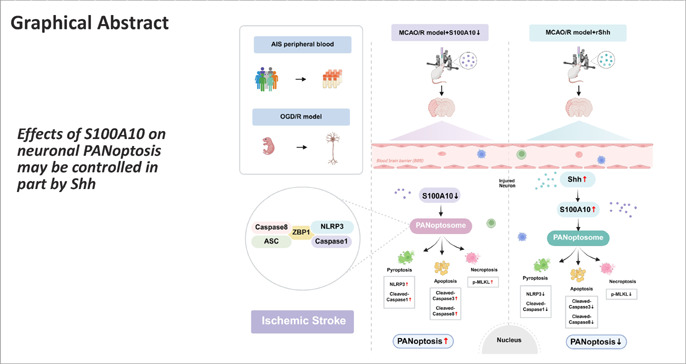

## Introduction

Ischemic stroke poses a substantial threat to human life and is a leading cause of global disability and mortality (Feigin et al., 2018; Gorelick, 2019; Ding et al., 2022; Tuo et al., 2022a). Currently, the primary clinical treatments for acute ischemic stroke are intravenous alteplase and thrombectomy (Catanese et al., 2017), which are relatively effective. However, some patients still experience poor outcomes owing to the narrow therapeutic window and the complications of cerebral ischemia–reperfusion injury (Mao et al., 2022). Therefore, novel treatment strategies are urgently needed.

Cerebral ischemia leads to neuronal death (Li et al., 2026). Previous studies have identified forms of neuronal cell death, including necrosis, apoptosis, and pyroptosis, which are critical determinants of neural function recovery after cerebral infarction and have been extensively investigated. PANoptosis, a novel programmed cell death proposed by Malireddi in 2019, is associated with the essential traits of apoptosis, pyroptosis, and necroptosis (Malireddi et al., 2019; Wang and Kanneganti, 2021; Gao et al., 2024). PANoptosis is characterized as a form of natural immune, lytic, and inflammatory cell death, propelled by caspases and receptor-interacting protein kinases (RIPKs), and regulated by PANoptosome assemblies (Pandeya and Kanneganti, 2024). It has been implicated in many diseases, such as autoimmune disorders, neurodegenerative diseases, cerebrovascular conditions, malignancies, infections, metabolic disorders, and retinal ischemia-reperfusion injury (Yan et al., 2023; Lan et al., 2024; Sun et al., 2024; Zhang et al., 2024b; Wan et al., 2026). Our preliminary studies have also demonstrated the occurrence of PANoptosis in neurons following ischemic stroke (Tang et al., 2025). Therefore, studying how to regulate PANoptosis may help identify new targets or strategies for treating cerebral ischemic stroke.

S100 calcium-binding protein A10 (S100A10), also known as p11 (Rescher and Gerke, 2008), is a member of the S100 protein family. Localized on chromosome 1q21 (Wang et al., 2023), it can bind to Annexin A2 to form heterotetrameric complex (S100A10)2–(AnnexinA2)2 (O’Connell et al., 2010). Unlike other family proteins, the S100A10 protein has calcium-binding protein mutation sites and is not sensitive to calcium ions (Seo and Svenningsson, 2020; Głowacka et al., 2021). S100A10 is extensively dispersed in the body (Saris et al., 1987). In the brain, S100A10 is predominantly found within neurons (Milosevic et al., 2017) and is also expressed in A2 reactive astrocytes (Fujita et al., 2018; Seo and Svenningsson, 2020). It is closely associated with a variety of central nervous system disorders, including neoplasms, neurodegenerative diseases, psychiatric disorders, and cerebrovascular diseases (Svenningsson et al., 2006; Green et al., 2017; Chédeville et al., 2020; Guo et al., 2021). Within the context of cerebrovascular diseases, research has predominantly focused on astrocytes (Hernández et al., 2021). As a result, it remains unclear whether and how S100A10 modulates neuronal PANoptosis and impacts outcomes following ischemic brain injury.

The sonic hedgehog (Shh) signaling pathway plays a role in tissue repair and regeneration following ischemic brain damage (Liu et al., 2018). Previous studies have demonstrated that Shh exerts neuroprotective effects in ischemic brain injury by suppressing apoptotic pathways, promoting tissue repair, and modulating neuroinflammatory responses (Huang et al., 2014; Cheng et al., 2015; Tang et al., 2017; Yu et al., 2017; Yang et al., 2024). S100A10 is closely associated with neurogenesis, modulating the neuroprotective effects against BDNF-mediated neurotoxicity and enhancing synaptic plasticity (Egeland et al., 2010; Park et al., 2016). In addition, p11 interacts with the Bcl-xL/Bcl-2-associated death promoter and inhibits its pro-apoptotic activity, suggesting that p11 could play a role in suppressing apoptosis (Hsu et al., 1997). The SHH/PI3K/Bcl-2 signaling pathway may play a critical role in protecting neurons from apoptosis induced by oxidative stress (Dai et al., 2012). However, it is not clear whether Shh signaling and S100A10 are involved in the regulation of neuronal PANoptosis after ischemic brain injury, and whether they interact with each other.

The aims of this study were to elucidate whether S100A10 and Shh influence PANapoptosis following ischemic brain injury, and whether there is a potential association between these two factors. The findings indicate that the role of S100A10 in regulating neuronal PANoptosis may be at least partially modulated by Shh.

## Methods

### Clinical samples

Serum samples were collected from 66 patients diagnosed with acute ischemic stroke (AIS) and 24 healthy controls at the Neurology Department of the First Affiliated Hospital of Chongqing Medical University between August 1, 2023 and February 29, 2024.

Brain tissue was collected from the core infarction area of the right temporal lobe in a AIS patient with a large right hemispheric infarction who underwent decompressive craniectomy at the Neurosurgery Department of the Third Hospital of Mianyang between October 1, 2023 and April 30, 2024.

Inclusion criteria for the AIS group were (1) age ≥ 18 years, (2) diagnosis of AIS by a neurologist (Sacco et al., 2013), (3) radiologically confirmed acute ischemic stroke via computed tomography or magnetic resonance imaging, and (4) serum sample collection completed within 72 hours of stroke onset. Criteria for the Healthy Control Group were age-matched healthy individuals with no history of stroke or neurological disorders (*n* = 24).

Exclusion criteria were as follows: (1) history of hemorrhagic stroke, (2) significant neurological disorder (e.g., Parkinson’s disease, Alzheimer’s disease), (3) severe craniocerebral trauma, (4) severe inflammatory or autoimmune diseases, (5) major surgery within the preceding 6 months, (6) chronic use of anticoagulants or immunosuppressive agents, and (7) incomplete clinical records or follow-up data.

Before centrifugation at 3000 × *g* for 15 minutes, blood samples were incubated at room temperature for 30 minutes. Serum aliquots were stored at –80°C. Serum S100A10 levels were quantified using a commercial enzyme-linked immunosorbent assay (ELISA; Meimian, Jiangsu, China, Cat# MM-50547H1) kit. Fresh brain tissue (approximately 1.5 cm × 1.7 cm × 1.3 cm) from surgical resection was rinsed with normal saline, fixed immediately in 4% paraformaldehyde (PFA), and processed into paraffin-embedded sections for subsequent analysis.

The use of human peripheral blood samples was approved by the Ethics Committee of the First Affiliated Hospital of Chongqing Medical University (No. K2023-270) on July 11, 2023. The use of human brain tissue was approved by the Ethics Committee of the Third Hospital of Mianyang and the First Affiliated Hospital of Chongqing Medical University (No. K2023-270) on July 11, 2023. This study was conducted in accordance with the *Declaration of Helsinki*. Written informed consent was obtained from all participants.

### Primary cortical neuron culture

Primary cortical neurons were isolated from newborn Sprague–Dawley (SD) rats within 24 hours of birth, as previously described (Yu et al., 2017). Culture plates were coated with poly-D-lysine (Solarbio, Beijing, China, Cat# P2100) solution for 2 hours, air-dried, and used within 24 hours. Neonatal SD rats were euthanized by excessive exposure to carbon dioxide, disinfected with 75% ethanol, decapitated on ice, and their brains were rapidly extracted. Meninges and noncortical tissues were carefully removed, and the remaining cortical tissue was minced with sterile ophthalmic scissors. Tissue fragments were digested in 0.125% trypsin-EDTA (Beyotime, Shanghai, China, Cat# C0201) at 37°C for 15 minutes with gentle agitation. Digestion was terminated by adding Dulbecco’s Modified Eagle Medium (DMEM; Gibco, Beijing, China, Cat# 11965092) supplemented with 10% fetal bovine serum (Gibco, Cat# 10099158). The cell suspension was triturated gently, filtered through a 40-μm strainer, and centrifuged at 1000 × *g* for 5 minutes. Pelleted cells were resuspended and seeded onto precoated plates in DMEM containing 10% fetal bovine serum. After 4 hours, the medium was replaced with neuron-specific complete medium (Neurobasal medium, Gibco, Cat# 10888022) supplemented with 2% B-27 (Gibco, Cat# A3582801), 1% penicillin-streptomycin (Beyotime, Cat# C0222), and 5 mM L-glutamine (Gibco, Cat# 25030081). Half-medium changes were performed every 48 hours, and neurons were cultured for 7 days before further experimentation.

### Oxygen‒glucose deprivation/reoxygenation model

An *in vitro* neuron oxygen‒glucose deprivation/reoxygenation (OGD/R) model was constructed after neuronal culture (Tang et al., 2017). In brief, primary cortical neurons were washed twice with phosphate-buffered saline (PBS; Biosharp, Shanghai, China, Cat# BL316A) and incubated in Hank’s Balanced Salt Solution (D-hanks; Biosharp, Cat# BL559A). Neurons were then transferred to a hypoxia chamber (Thermo Fisher, Waltham, MA, USA) maintained at 94% N_2_, 5% CO_2_, and 1% O_2_ conditions for 4 hours. Untreated control neurons were cultured in normal D-Hanks under normoxic conditions. After OGD/R, the D-hanks solution was aspirated, and neurons were replenished with fresh neuronal complete medium for reoxygenation.

### Animals

To avoid the influence of estrogen on ischemic damage and outcomes, this study used only male rats (Shi et al., 2001; Koellhoffer and McCullough, 2013). Adult male (8 weeks old, 200–250 g) and neonatal SD rats were obtained from the Chongqing Medical University’s Animal Experiment Department (license No. SCXK (Yu) 2022-0010). Rats were randomly assigned to six groups (*n* = 30/group): Sham group, middle cerebral artery occlusion/reperfusion (MCAO/R) group, MCAO/R + Ad-NC group, MCAO/R + Ad-shS100A10 group, MCAO/R + rShh group, and MCAO/R + Ad-shS100A10 + rShh group. Animals were housed in a specific pathogen-free facility under controlled temperature (22 ± 1°C) and humidity (50% ± 5%) conditions, and a 12-hour light/dark cycle (light on 09:00–21:00), with *ad libitum* access to food and water. All procedures complied with the guidelines of the Animal Ethics Committee of Chongqing Medical University (approval No. 2021-559 on November 23, 2021), and strictly adhered to the Animal Research: Reporting of *In Vivo* Experiments (ARRIVE) Guidelines (Percie du Sert et al., 2020). Outcome assessments were performed by blinded investigators under masked experimental conditions.

### Middle cerebral artery occlusion/reperfusion model

A rat model of MCAO/R was established as previously described (Yu et al., 2021). Briefly, rats were anesthetized with 2,2,2-tribromoethanol (1.25%, 10 mL/kg intraperitoneally; Lab Anim Tech, Beijing, China). The right middle cerebral artery underwent 120-minute occlusion, after which the filament was withdrawn to initiate reperfusion. Rats with Longa scores (Longa et al., 1989) of 1–3 were included in subsequent analyses. Sham-operated rats underwent identical surgical procedures, except for filament insertion. Body temperature was maintained using a heating pad throughout the surgery.

### Intracerebroventricular catheterization

Intracerebroventricular catheterization was performed as previously described (DeVos and Miller, 2013). Rats were anesthetized with 2,2,2-tribromoethanol (1.25%, 10 mL/kg ip; Lab Anim Tech) and secured in a stereotaxic apparatus (RWD Life Science, Shenzhen, China). Subsequently, a single 23-gauge guide cannula (RWD Life Science) was inserted into the right lateral ventricle at the following coordinates: 0.9 mm posterior, 3.6 mm ventral, 1.5 mm lateral (Yang et al., 2018). The guide cannula was fixed with dental cement and sealed with a stainless-steel wire to prevent blockage.

### *In vivo* adenovirus injection and treatment

Adenoviruses for S100A10-shRNA (Ad-shS100A10) and negative control (Ad-NC) were synthesized by GenePharma (Shanghai, China). Four days before the commencement of MCAO/R, adenovirus was administered into the right lateral ventricle (5 µL/rat). The efficacy of S100A10 knockdown was verified via western blot analysis.

### Lentiviral transduction of cortical neurons

Lentiviruses shNC and shS100A10 were purchased from GenePharma. Primary cortical neurons were infected at a multiplicity of infection of 10 after 7 days of culture *in vitro*. Within 24 hours, the culture medium was substituted, and the cells were incubated for an additional 2 days before the OGD/R treatment.

### Drug administration

For *in vivo* experiments, SD rats were treated with MCAO/R (after reperfusion for 2 hours) and then PBS with 5 µL recombinant Shh (rShh; MedChemExpress, Monmouth Junction, NJ, USA, Cat# HY-P7409) by right lateral ventricle injection.

For *in vitro* experiments, primary cortical neurons underwent 4 hours of OGD followed by treatment with PBS with 10 µM rShh for 24 hours.

### Cerebral infarct volume

Infarct volume was quantified 3 days after MCAO/R using 2,3,5-triphenyl tetrazolium chloride (TTC) staining (Solarbio, Cat# G3005) as previously described (Yu et al., 2017). Rats were euthanized by carbon dioxide overexposure, and rat brains were quickly extracted, frozen at –20°C for 20 minutes, and then cut into 5 coronal slices (2 mm). The sections were incubated in TTC solution at 37°C for 15 minutes, followed by fixation in 4% PFA at 4°C for 2 hours. High-resolution images of the slices were collected, and the infarction area was calculated using ImageJ software (version 1.8.0, National Institutes of Health, Bethesda, MD, USA).

### Neurological function score

Neurological deficits were assessed 3 days after stroke by a blinded investigator using the Longa score (Longa et al., 1989), modified Neurological Severity Score (mNSS) (Yu et al., 2017), and modified Bederson Score (Bederson et al., 1986).

The Longa scoring scale was as follows: 0 points, asymptomatic; 1 point, when lifting the tail, the contralateral forelimb cannot be straightened due to injury; 2 points, circling toward the injured side; 3 points, falling to the opposite side; 4 points, unable to walk spontaneously and unconscious. Animals with a Longa score of 1–3 were used for the animal model.

Bederson’s neurological symptom classification was as follows: Grade 0, asymptomatic; Grade 1, when lifting the tail, the contralateral forelimb cannot be straightened; Grade 2, flexion of the contralateral forelimb of the injury, without circling behavior; Grade 3, flexion of the contralateral forelimb, accompanied by spontaneous circling behavior.

The mNSS is a comprehensive scale for evaluating neurological function in rats, including motor function, sensory function, reflex activity, and balance ability. It provides an effective assessment of the severity of neural impairment, with a total score of 18 points. A higher score indicates more severe neurological damage.

### Western blot assay

Primary cortical neurons and rat brain tissue were lysed using radioimmunoprecipitation assay buffer (Beyotime, Cat# P0013E) containing protease/phosphatase inhibitor cocktails (Beyotime, Cat# ST505). Proteins extracted from tissues or cells were separated on 10%–12.5% SDS-polyacrylamide gels by electrophoresis (Beyotime, Cat# P0012A). Proteins were transferred to polyvinylidene difluoride membranes (Millipore, Burlington, MA, USA, Cat# IPVH00010). Membranes were blocked with 5% nonfat milk for 60 minutes and subsequently incubated overnight at 4°C with primary antibodies. After washing, membranes were incubated with an appropriate secondary antibody (1:5000 dilution) at room temperature for 1 hour. Protein bands were detected using Pierce ECL western blotting substrate (Zenbio, Chengdu, China, Cat# 17047). Band intensities were quantified using Image Lab 6.1 (Bio-Rad Laboratories, Hercules, CA, USA) and ImageJ software by a blinded investigator. The primary antibodies used in this study were anti-S100A10 (1:500, Proteintech, Wuhan, China, Cat# 11250-1-Ap), anti-Shh (1:1000, Proteintech, Cat# 20697-1-AP, RRID: AB_10694828), anti-nucleotide-binding domain-like receptor protein 3 (NLRP3; 1:1000, Abcam, Cambridge, UK, Cat# ab270449), anti-caspase 1 (1:1000, Proteintech, Cat# 22915-1-AP, RRID: AB_2876874), anti-caspase 3 (1:1000, CST, Danvers, MA, USA, Cat# 9662), anti-caspase 8 (1:1000, CST, Cat# 4927), anti-phospho-mixed lineage kinase domain-like (MLKL)(pSer345) (1:1000, Abcam, Cat# ab196436), anti-Z-DNA binding protein 1 (ZBP1; 1:500, Santa Cruz Biotechnology, Dallas, TX, USA, Cat# sc-271483), anti-apoptosis-associated speck-like protein (ASC; 1:1000, Proteintech, Cat# 10500-1-AP, RRID: AB_2174862), anti-glyceraldehyde phosphate dehydrogenase (GAPDH; 1:5000, Proteintech, Cat# 10494-1-AP, RRID: AB_2263076), and anti-β-actin (1:5000, Proteintech, Cat# 66009–1-Ig, RRID: AB_2687938). The secondary antibodies used were horseradish peroxidase-conjugated goat anti-rabbit IgG (1:5000, Proteintech, Cat# SA00001-2, RRID: AB_2722564) and goat anti-mouse IgG (1:5000, Proteintech, Cat# SA00001-1, RRID: AB_2722565).

### Immunofluorescence staining and terminal deoxynucleotidyl transferase dUTP nick-end labeling assay

During the preparation of paraffin sections, rats were anesthetized with 2,2,2-tribromoethanol (1.25%, 10 mL/kg intraperitoneally; Lab Anim Tech, Beijing, China). Cardiac perfusion was performed with 250 mL of saline and 250 mL of 4% PFA. Brain tissues were then collected and placed in 4% PFA overnight, followed by dehydration and paraffin embedding. Subsequently, continuous coronal sections (4 μm thickness) were obtained.

Cells were fixed in 4% formaldehyde solution (Biosharp, Cat# BL539A) for exactly 10 minutes. For preparation of tissue sections, each slide underwent deparaffinization and rehydration following standard protocols. Then, cells and tissue were permeabilized with 0.3% Triton X-100 (Solarbio, Cat# T8200) for 10 minutes, followed by antigen retrieval in Tris-Ethylene Diamine Tetraacetic Acid solution (Solarbio, Cat# C1038). Nonspecific binding was blocked with 10% normal goat serum (Beyotime, Cat# C0265) for 1 hour. Subsequently, they were incubated with specified primary antibodies at 4°C and with secondary antibodies for 1 hour at 37°C. Nuclei were counterstained with 4′,6-diamidino-2-phenylindole (DAPI; Beyotime, Cat# C1005). Images were acquired using a fluorescence microscope (Andor, Oxford, UK). ImageJ software was used to quantitatively analyze the selected regions of interest in each brain slice and the number or fluorescence intensity of positive cells in the cell smears. The primary antibodies used were rabbit anti-S100A10 (Proteintech, Cat# 11250-1-Ap, RRID: AB_2269906, 1:200), rabbit anti-NLRP3 (Abcam, Cat# ab270449, 1:200), rabbit anti-caspase 1 (Proteintech, Cat# 22915-1-AP, RRID: AB_2876874, 1:200), rabbit anti-caspase 3 (Proteintech, Cat# 19677-1-AP, RRID: AB_10733244, 1:200), rabbit anti-MLKL (Proteintech, Cat# 21066-1-AP, RRID: AB_2878804, 1:200), mouse anti-neuronal nuclei (NeuN; Proteintech, Cat# 66836-1-Ig, RRID: AB_2882179, 1:200), mouse anti-glial fibrillary acidic protein (GFAP; Proteintech, Cat# 60190-1-Ig, RRID: AB_10838694, 1:200), mouse anti-ionized calcium binding adaptor molecule 1 (Iba1; Abcam, Cat# ab283319, 1:200), and mouse anti-microtubule-associated protein 2 (MAP2; Thermo Fisher, Cat# 13-1500, RRID: AB_2533001, 1:200). The secondary antibodies used were goat anti-mouse IgG Dylight 594 (1:200, Abbkine, Wuhan, China, Cat# A23410), goat anti-rabbit IgG Dylight 488 (1:200, Abbkine, Cat# A23220), and polymer-horseradish peroxidase anti-rabbit secondary antibody (AFIHC003, Hunan, China).

Apoptosis in paraffin-embedded brain tissue sections was assessed using the terminal deoxynucleotidyl transferase dUTP nick-end labeling (TUNEL) assay (Servicebio, Wuhan, China, Cat# G1504) according to the manufacturer’s protocol. In brief, deparaffinized and rehydrated sections were treated with proteinase K, incubated with TUNEL reaction solution, and counterstained with DAPI. Fluorescence images were captured using a fluorescence microscope (Andor). TUNEL-positive cells were quantified using ImageJ.

### Co-immunoprecipitation

Protein interactions were validated by co-immunoprecipitation (Co-IP). Initially, primary cortical neuron lysates were incubated overnight at 4°C with anti-ASC (1:1000, Proteintech, Cat# 10500-1-AP, RRID: AB_2174862) or mouse IgG (2.5 µg, Beyotime, Cat# A7028) under gentle rotation. On the second day, protein A/G magnetic beads (Bimake, Houston, Texas, USA) were then added to the lysate and incubated for 2 hours at 4°C. After washing, bound proteins were eluted by boiling in 2× SDS loading buffer for 10 minutes and analyzed by western blotting.

### Cell viability

Cell viability was assessed using Cell-Counting Kit 8 (CCK-8; Beyotime, Cat# C0037) according to the manufacturer’s protocol. Primary cortical neurons were seeded onto 96-well plates. After viral transfection or treatment, CCK-8 solution was added to each well, followed by incubation at 37°C for 1–4 hours. Cell viability was assessed through measurement of the optical density at 450 nm. Percent of control served as the metric for assessing cell viability.

### Enzyme-linked immunosorbent assay

Levels of S100A10 in human serum samples were analyzed using ELISA kits (Meimian, Cat# MM-50547H1), strictly adhering to the manufacturer’s protocols.

### Statistical analysis

All data were expressed as the mean ± standard deviation (SD). GraphPad Prism software (version 9.5.0; San Diego, CA, USA, http://www.graphpad.com) was used to perform statistical analyses. The Shapiro–Wilk test was used to assess data normality. For comparisons between two groups, a two-tailed Student’s *t*-test was applied. One-way analysis of variance followed by Tukey’s *post hoc* test was used for multiple-group comparisons. Statistical significance was defined as *P* < 0. 05.

## Results

### S100 calcium-binding protein A10 expression is increased in patients with acute ischemic stroke, middle cerebral artery occlusion/reperfusion rats, and oxygen and oxygen‒glucose deprivation/reoxygenation neurons

**Figure 1:**
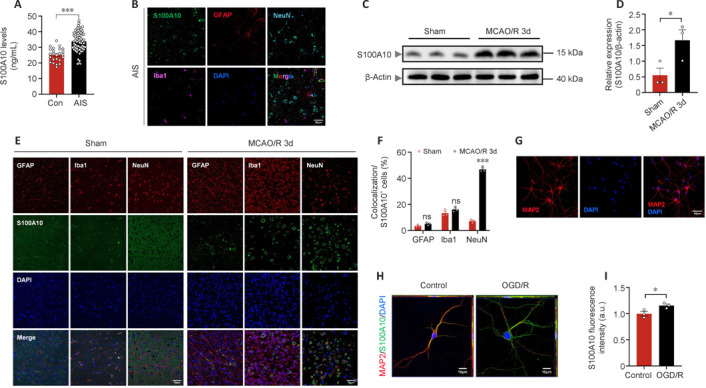
Cerebral ischemia upregulated S100A10 expression. (A) Relative serum S100A10 levels in patients with AIS and healthy controls (*n* = 24) within 3 days (*n* = 66), as measured by ELISA. (B) Representative immunofluorescence staining of S100A10 (green), GFAP (red), Iba1 (violet), NeuN (turquoise), and DAPI (nuclei, blue) in the infarct core region of the right temporal lobe in patients with AIS within 3 days of onset. Scale bar: 50 μm. (C, D) S100A10 protein expression in rat brain samples three days post-MCAO/R injury through Western blot analysis (*n* = 3). (E) Immunofluorescence staining assessing S100A10 (green) expression across various cell types (red), including Iba1^+^ microglia, NeuN^+^ neurons, and GFAP^+^ astrocytes, in the brains of rats subjected to sham or MCAO/R for 3 days. DAPI (blue) was used to stain the cellular nuclei. Scale bars: 50 μm. (F) Quantification of the colocalization of NeuN, GFAP, or Iba1 with S100A10 in the sham and MCAO/R groups (*n* = 3). (G) Identification of neurons (purity > 90%). Scale bar: 50 μm. (H) Z-stack maximum intensity projection of primary cortical neurons. The blue color (DAPI) highlights the cell nuclei. Scale bars: 10 μm. (I) Immunofluorescence intensity of S100A10. Data are expressed as the mean ± SD. All experiments were independently repeated at least three times. *P < 0.05, ***P < 0.001, *vs.* sham or control group (two-tailed Student’s *t*-test). AIS: Acute ischemic stroke; Con: control; DAPI: 4′,6-diamidino-2-phenylindole; ELISA: enzyme-linked immunosorbent assay; GFAP: glial fibrillary acidic protein; Iba-1: ionized calcium-binding adapter molecule 1; MAP2: microtubule-associated protein 2; MCAO/R: middle cerebral artery occlusion/reperfusion; NeuN: neuronal nuclei marker; ns: not significant; S100A10: S100 calcium-binding protein A10.

In serum samples from patients with AIS within 3 days of injury, ELISA showed significantly elevated S100A10 levels compared with those in healthy controls (**Figure 1A**). Immunofluorescence analysis confirmed predominant S100A10 expression in neurons of patients with AIS (**Figure 1B**). Similarly, in the MCAO/R rat model, S100A10 protein expression was increased in ischemic brain tissue at 3 days after reperfusion compared with that in sham-operated controls (**Figure 1C** and **D**). S100A10 was colocalized with almost all neurons (NeuN), some microglia (Iba1), and few astrocytes (GFAP) in ischemic brain tissue (**Figure 1E** and **F**). Neuronal purity in cultures exceeded 90%, as confirmed by immunofluorescence staining for the neuronal marker MAP2 (**Figure 1G**). Z-stack imaging showed a robust spatial overlap of MAP2 and S100A10 in neuronal cell bodies and dendrites (**Figure 1H**). Furthermore, S100A10 fluorescence intensity was increased after OGD/R treatment compared with control (**Figure 1H** and **I**).

Taken together, these results from patients with AIS, MCAO/R rats, and OGD/R neurons indicated that S100A10 expression was significantly increased following ischemic brain injury, with neurons constituting the primary cellular source of S100A10.

### Middle cerebral artery occlusion/reperfusion injury activates PANoptosis

**Figure 2:**
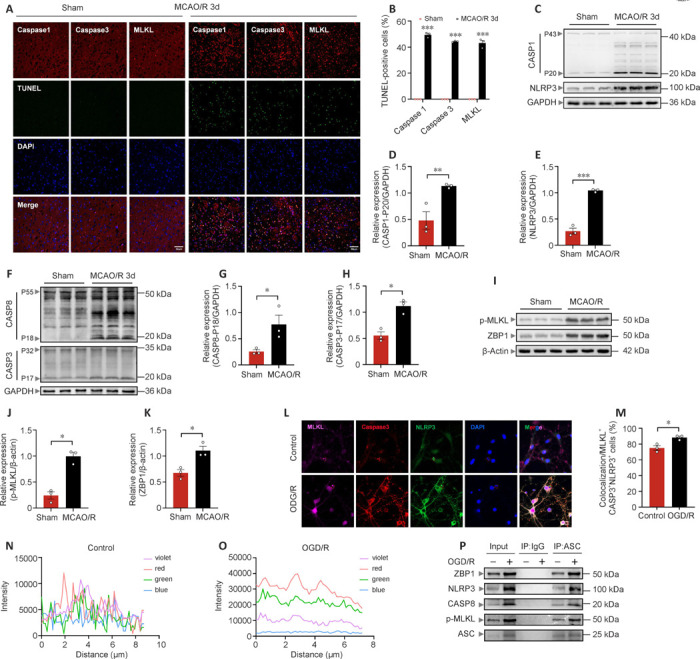
Cerebral ischemia induced PANoptosis (A) Representative images of 3-day post-MCAO/R rats stained with caspase-1 (red), caspase-3 (red), MLKL (red), and DAPI (blue) in combination with TUNEL (green). Scale bars: 50 μm. (B) Quantitative analysis of TUNEL-positive cells. (C–K) Western blot analysis of (C–E) NLRP3, pro- (p43) and cleaved caspase-1 (CASP1, p20); (F–H) pro- (p55) and cleaved caspase-8 (CASP8, p18), pro- (p32) and cleaved caspase-3 (CASP3, p17); and (I–K) p-MLKL and ZBP1 in the sham and MCAO/R groups (*n* = 3). (L) Immunofluorescence staining of NLRP3 (green), Caspase3 (red), MLKL (violet), and DAPI (nuclei, blue) in primary cortical neurons after OGD/R. Scale bar: 20 μm. (M) Quantification of MLKL^+^Caspase3^+^NLRP3^+^ cells. (N, O) Line plots of Caspase1, Caspase3, and MLKL fluorescence signal intensities along the red arrow in L. (P) Interactions between ZBP1, NLRP3, CASP8, p-MLKL, and ASC in primary cortical neurons after OGD/R were elucidated through Co-IP assay. Data are expressed as the mean ± SD. All experiments were independently repeated at least three times. *P < 0.05, **P < 0.01, ***P < 0.001, *vs.* sham group (two-tailed Student’s *t*-test). ASC: Apoptosis-associated speck-like protein containing a CARD; CASP1: cysteinyl aspartate specific protease 1, caspase1; CASP3: cysteinyl aspartate specific protease 3, caspase3; CASP8: cysteinyl aspartate specific protease 8, caspase8; DAPI: 4′,6-diamidino-2-phenylindole; GAPDH: glyceraldehyde phosphate dehydrogenase; MCAO/R: middle cerebral artery occlusion/reperfusion; MLKL: mixed lineage kinase domain-like protein; NLRP3: nucleotide-binding domain-like receptor protein 3; OGD/R: oxygen‒glucose deprivation/reperfusion; p-MLKL: phosphorylated mixed lineage kinase domain-like protein; TUNEL: terminal deoxynucleotidyl transferase-mediated dUTP nick-end labeling; ZBP1: Z-DNA binding protein 1.

Neuronal death is a hallmark of ischemic stroke pathology (Yuan, 2009; Hoque et al., 2016). Next, we detected whether PANoptosis is present in ischemic brain tissue. We determined the proportion of TUNEL^+^ (caspase1^+^/caspase3^+^/MLKL^+^) cells from the total number of cell nuclei through TUNEL staining and double immunofluorescence. The results showed apoptotic cells colocalized with markers of pyroptosis (caspase1), apoptosis (caspase3), and necroptosis (MLKL) in ischemic regions, indicating concurrent activation of multiple cell death pathways (**Figure 2A** and **B**). Western blot analysis showed the upregulation of PANoptosis-associated proteins, including ZBP1, NLRP3/cleaved caspase-1 (pyroptosis), cleaved caspase-3/caspase-8 (apoptosis), and p-MLKL (necroptosis), in ischemic tissue compared with controls (**Figure 2C–K**).

To confirm PANoptosome complex formation—a hallmark of PANoptosis—we performed Co-IP and colocalization studies. Immunofluorescence showed significant colocalization of NLRP3, cleaved caspase-3, and MLKL in neurons following OGD/R, with fluorescence intensity significantly higher than that in untreated neurons (**Figure 2L–O**). Co-IP assay in OGD/R-treated neurons showed direct interactions between ASC (a pyroptosis adaptor) and ZBP1, NLRP3, CASP8, and p-MLKL, confirming PANoptosome assembly (**Figure 2P**).

Collectively, these findings provide compelling evidence that ischemic brain injury triggers PANoptosis, characterized by coordinated activation of pyroptotic, apoptotic, and necroptotic machinery and PANoptosome complex formation.

### Knockdown of S100 calcium-binding protein A10 aggravates neuronal PANoptosis after oxygen‒glucose deprivation/reoxygenation injury *in vitro*

**Figure 3:**
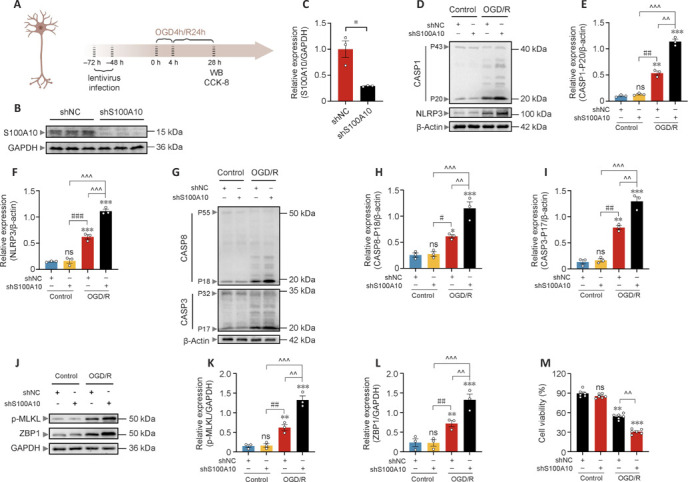
Knockdown of S100A10 aggravated neuronal PANoptosis after OGD/R injury *in vitro.* (A) The experimental procedure. (B, C) Western blot detection of S100A10 protein levels in neurons transfected with S100A10 shRNA. (D–L) Western blot analysis of PANoptosis-associated proteins in the control and OGD/R groups. (M) Cell viability was determined using the CCK-8 assay. Data are expressed as the mean ± SD. All experiments were independently repeated at least three times. *P < 0.05, **P < 0.01, ***P < 0.001, *vs*. shNC group or Control + shNC group; #*P* < 0.05, ##*P* < 0.01, *vs.* control + shS100A10 group; ^*P* < 0.05, ^^*P* < 0.01, ^^^*P* < 0.001, *vs*. OGD/R + shS100A10 group (two-tailed Student’s *t*-test [C] or one-way analysis of variance followed by Tukey’s multiple comparison test [E, F, H, I, K–M]). CASP1: Cysteinyl aspartate specific protease 1, caspase 1; CASP3: Cysteinyl aspartate specific protease 3, caspase 3; CASP8: Cysteinyl aspartate specific protease 8, caspase 8; CCK-8: Cell Counting Kit-8; GAPDH: glyceraldehyde phosphate dehydrogenase; NLRP3: Nucleotide-binding domain-like receptor protein 3; ns: not significant; OGD/R: oxygen–glucose deprivation/reperfusion; p-MLKL: phosphorylated mixed lineage kinase domain-like protein; S100A10: S100 calcium-binding protein A10; WB: western blot; Z-DNA binding protein 1.

The results from patients with AIS, MCAO/R rats, and OGD/R neurons suggested that cerebral ischemic injury upregulated S100A10 expression and activated PANoptosis, particularly in neurons. We next investigated whether S100A10 plays a role in PANoptosis. Therefore, neuronal S100A10 *in vitro* was knocked down by lentiviral transduction to investigate the effect on PANoptosis (**Figure 3A**). As shown in **Figure 3B** and **C**, S100A10 was successfully silenced in primary rat cortical neurons. Western blot analysis showed that S100A10 knockdown increased PANoptosis-related proteins in OGD/R-treated neurons compared with the OGD/R+sh-NC group, including pyroptosis markers (cleaved-caspase1 (p20), NLRP3), apoptosis markers (cleaved-caspase3 (p17), cleaved-caspase8 (p18)), and necroptosis markers (p-MLKL), and the PANoptosis sensor ZBP1 (**Figure 3D–L**). Additionally, CCK-8 assays indicated that S100A10 knockdown markedly reduced neuronal viability post-OGD/R (**Figure 3M**). These results confirmed that S100A10 knockdown exacerbated PANoptosis and decreased survival in neurons after OGD/R *in vitro*.

### Recombinant Shh upregulates S100A10 expression and inhibits PANoptosis

**Figure 4:**
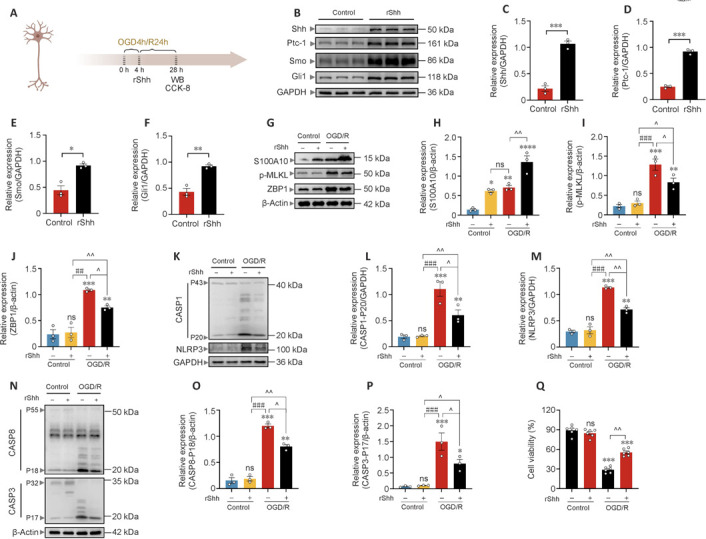
Recombinant Shh affected neuronal PANoptosis and S100A10 protein expression after OGD/R injury *in vitro*. (A) The experimental procedure. (B–F) Relative expression of Shh, Ptc-1, Smo, and Gli1 in primary cortical neurons after rShh treatment, as determined by Western blotting. (G–P) Western blot analysis was used to assess the levels of PANoptosis-related proteins in the control and OGD/R groups. (Q) Neuronal viability was evaluated using the CCK-8 assay. Data are expressed as the mean ± SD. All experiments were independently repeated at least three times. *P < 0.05, **P < 0.01, ***P < 0.001, ****P < 0.0001, *vs*. control group; #*P* < 0.05, ##*P* < 0.01, *vs*. Control + rShh group; ^*P* < 0.05, ^^*P* < 0.01, ^^^*P* < 0.001, *vs.* OGD/R+rShh group (two-tailed Student’s *t* test [C–F] or one-way analysis of variance followed by Tukey’s multiple comparison test [H‒J, L, M, O‒Q]). CASP1: Cysteinyl aspartate specific protease 1, caspase 1; CASP3: cysteinyl aspartate specific protease 3, caspase 3; CASP8: cysteinyl aspartate specific protease 8, caspase 8; CCK-8: Cell Counting Kit-8; GAPDH: glyceraldehyde phosphate dehydrogenase; Gli1: Glioma-associated oncogene 1; NLRP3: nucleotide-binding domain-like receptor protein 3; ns: not significant; OGD/R: oxygen‒glucose deprivation/reperfusion; p-MLKL: phosphorylated mixed lineage kinase domain‒like protein; Ptc-1: patched-1; rShh: recombinant Shh; S100A10: S100 calcium-binding protein A10; Shh: Sonic Hedgehog; Smo: smoothened; WB: Western blot; ZBP1: Z-DNA binding protein 1.

We next analyzed whether rShh affects S100A10 expression and PANoptosis after neuronal OGD/R *in vitro* (**Figure 4A**). Shh, Smo, and Gli1 are important components of the Shh pathway (Huang et al., 2014). Shh, Ptc-1, Smo, and Gli1 levels were elevated in the rShh group compared with the control group (**Figure 4B–F**). Western blot analysis showed that rShh enhanced S100A10 protein levels (**Figure 4G** and **H**) and suppressed PANoptosis-associated proteins, including pyroptosis (cleaved-Caspase1 (p20), NLRP3), apoptosis (cleaved-Caspase3 (p17), cleaved-Caspase8 (p18)) and necroptosis (p-MLKL) proteins, and the PANoptosis sensor ZBP1 (**Figure 4I–P**). Moreover, rShh significantly increased neuronal survival (**Figure 4Q**). These data suggested that rShh upregulated S100A10, alleviated PANoptosis, and promoted neuronal survival after OGD/R *in vitro*.

### Recombinant sonic hedgehog reverses effects of S100 calcium-binding protein A10 knockdown on PANoptosis after middle cerebral artery occlusion/reperfusion injury in rats

**Figure 5:**
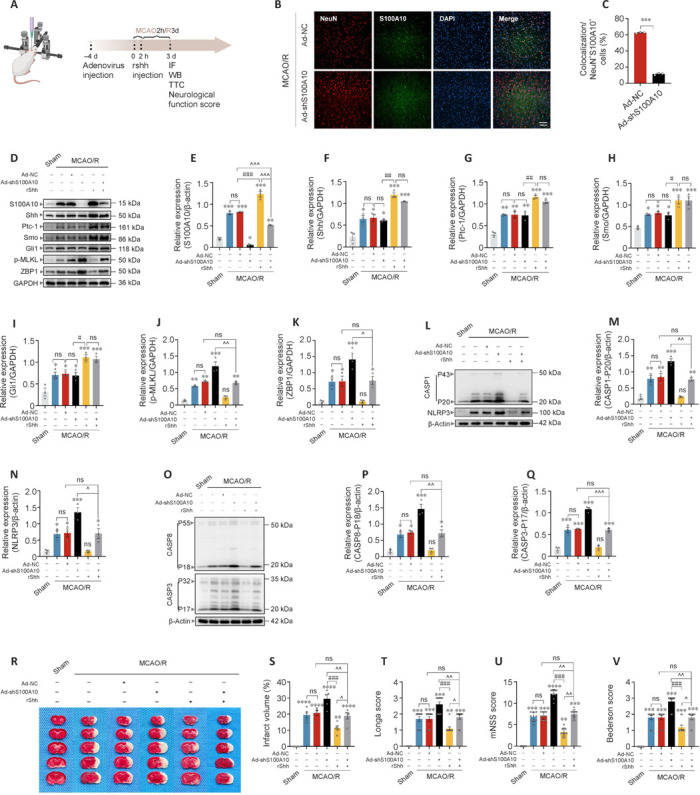
Recombinant Shh reversed the effects of S100A10 knockdown on PANoptosis after MCAO/R injury in rats. (A) The experimental procedure. (B) Immunofluorescence staining of S100A10 (green), NeuN (red), and DAPI (nuclei, blue) in the Ad-NC and Ad-shS100A10 groups. Scale bar: 100 μm. (C) Quantitative analysis of NeuN^+^S100A10^+^ cells. (D–Q) Western blot analysis of the expression of PANoptosis-related proteins in the sham and MCAO/R 3d groups (*n* = 3). (R, S) TTC staining was used to assess the infarct volume 3 days post-reperfusion (*n* = 5). While in R indicates infarct regions. (T–V) Neurological deficit assessments 3 days after MCAO/R injury (*n* = 10) were quantified through the Longa score (T), mNSS (U), and Bederson score (V). Data are expressed as the mean ± SD. All experiments were independently repeated at least three times. *P < 0.05, **P < 0.01, ***P < 0.001, *vs.* sham group; #*P* < 0.05, ##*P* < 0.01, ###*P* < 0.001, *vs.* MCAO/R + rShh group; ^*P* < 0.05, ^^*P* < 0.01, ^^^*P* < 0.001, *vs*. MCAO/R + Ad-shS100A10 + rShh group (two-tailed Student’s *t*-test [C] or one-way analysis of variance followed by Tukey’s multiple comparison test [E–K, M, N, P, Q, S–V]). CASP1: Cysteinyl aspartate specific protease 1, caspase 1; CASP3: cysteinyl aspartate specific protease 3, caspase 3; CASP8: cysteinyl aspartate specific protease 8, caspase 8; DAPI: 4’,6-diamidino-2-phenylindole; GAPDH: glyceraldehyde phosphate dehydrogenase; Gli1: glioma-associated oncogene 1; IF: immunofluorescence; MCAO/R: middle cerebral artery occlusion/reperfusion; NeuN: neuronal nuclei; NLRP3: nucleotide-binding domain-like receptor protein 3; ns: not significant; p-MLKL: phosphorylated mixed lineage kinase domain-like protein; Ptc-1: patched-1; rShh: recombinant Shh; S100A10: S100 calcium-binding protein A10; Shh: sonic hedgehog; Smo: smoothened; TTC: 2,3,5-triphenyl tetrazolium chloride; WB: western blot; ZBP1: Z-DNA binding protein 1.

We next investigated whether rShh affects PANoptosis via S100A10 in MCAO/R rats *in vivo* (**Figure 5A**). Western blot confirmed successful S100A10 knockdown in the Ad-shS100A10 group, and Shh, Ptc-1, Smo, and Gli1 were substantially elevated in the rShh group. Furthermore, compared with the MCAO/R group, rShh significantly upregulated S100A10 protein expression (**Figure 5D–I**). Immunofluorescence results showed that NeuN^+^S100A10^+^ cells were substantially reduced in the Ad-shS100A10 group compared with the Ad-NC group (**Figure 5B** and **C**). MCAO/R injury significantly increased PANoptosis-associated proteins compared with those in sham controls (**Figure 5D**, **J–Q**). Notably, S100A10 knockdown exacerbated PANoptosis, and rShh treatment reversed this effect (**Figure 5D**, **J–Q**). Additionally, S100A10 knockdown increased the infarct volume and impaired neurological function, and these changes were mitigated by rShh administration (**Figure 5R–V**). These results suggested that rShh rescued S100A10 deficiency-induced PANoptosis, attenuated brain damage, and improved functional recovery in an MCAO/R model.

## Discussion

In the present study, we showed that S100A10 levels were significantly elevated in the serum of patients with AIS, brain tissue of MCAO/R rats, and OGD/R neurons. MCAO/R and OGD/R injuries triggered PANoptosis activation. Knockdown of S100A10 exacerbated PANoptosis and upregulated PANoptosis-related proteins after MCAO/R and OGD/R injury. Recombinant Shh upregulated S100A10 expression, suppressed PANoptosis, and rescued the detrimental effects of S100A10 knockdown on PANoptosis and neurological outcomes after MCAO/R injury. These findings are the first to show that the effects of S100A10 on PANoptosis are regulated at least in part by Shh.

Numerous studies have shown that ischemic stroke is characterized by neuronal apoptosis (Love, 2003). The severity of ischemic stroke is predominantly determined by the extent of neuronal death in the infarct region (Ren et al., 2023). Previous studies have shown that nearly every type of regulatory necrosis plays a role in ischemic stroke development, including necroptosis (Li et al., 2019), pyroptosis (Tang et al., 2022; Li et al., 2023), ferroptosis (Tuo et al., 2022b), apoptosis (Liu et al., 2023), parthanatos, mitochondrial permeability transition pore-mediated necrosis (Li et al., 2020), and oncosis (Fricker et al., 2018; Ren et al., 2023). Pyroptosis, apoptosis, and necroptosis were initially viewed as distinct pathways, but emerging evidence has highlighted significant molecular crosstalk between these processes (Wang and Kanneganti, 2021; Cui et al., 2022). Consequently, the idea of comprehensive cellular demise was proposed, called PANoptosis, which is characterized by pyroptosis, apoptosis, and necroptosis, but cannot be attributed to any of them alone (Shi et al., 2024). PANoptosis has been linked to a spectrum of ailments, including infectious diseases (Zhou et al., 2023), cancer (Jiang et al., 2021), neurodegenerative disorders (Zeng et al., 2023), cerebrovascular diseases (Lan et al., 2024; Zhang et al., 2024b), and inﬂammatory conditions (Cui et al., 2022; Sun et al., 2024). To date, five types of PANoptosomes with distinct sensors and regulators have been characterized: ZBP1 PANoptosome, absent in melanoma 2 PANoptosome, RIPK1 PANoptosome, nucleotide-binding leucine-rich repeat receptor 12 PANoptosome, and NLR caspase recruitment domain-containing protein 5 PANoptosome (Qi et al., 2023; Sundaram et al., 2024). Of these, ZBP1 has been identified as a central regulator in PANoptosis activation (Karki and Kanneganti, 2023). Our previous and current studies indicate that AIS induces ZBP1 PANoptosome formation, promoting the occurrence of neuronal PANoptosis (Tang et al., 2025).

S100A10, a member of the calc-binding S100 protein family, plays multifunctional roles in physiological and pathological processes, including endocytosis, plasminogen activation, signal transduction, cell proliferation, and autophagy (Okura et al., 2023). In recent years, S100A10 has been associated with various pathophysiological processes, such as cancer, neurodegenerative disorders, and psychiatric diseases (Neyazi et al., 2018; Seo and Svenningsson, 2020). In glioblastoma, S100A10 is highly expressed and drives tumor progression by promoting plasmin-mediated extracellular matrix degradation, facilitating metastasis, invasion, proliferation, and drug resistance, and is significantly linked to poor outcomes in glioma patients (Chédeville et al., 2020; Qi et al., 2023). In a mouse model of amyotrophic lateral sclerosis, enhancing the function of the Sp1-p11-TASK-1 pathway results in excitatory toxicity affecting motor neurons. Reducing the expression of p11 was observed to extend lifespan within this model (Seo and Svenningsson, 2020). Svenningson et al. reported that S100A10-deficient mice exhibit depression-like behaviors, and that antidepressant treatment upregulates S100A10 expression in rodent brains, suggesting its role in mood regulation (Svenningsson et al., 2006). In addition, S100A10 exerts an antiapoptotic effect. It interacts with the Bcl-xL/Bcl-2-associated death promoter to inhibit their proapoptotic activities (Hsu et al., 1997). In Osa cells, S100A10 knockdown inhibits proliferation, migration, and invasion while promoting apoptosis via AKT/mTOR pathway-mediated glycolysis modulation (Okura et al., 2023). Similarly, S100A10 protects epithelial cells from apoptosis during apical stratification, underscoring its role in maintaining cellular survival (Ito et al., 2023).

S100A10 is widely distributed in the brain in various structures and cell types, but is mainly expressed in neurons (Milosevic et al., 2017) and type A2 reactive astrocytes (Fujita et al., 2018). Our study showed that S100A10 was predominantly colocalized with neurons and less colocalized with astrocytes, consistent with previous reports (Milosevic et al., 2017; Fujita et al., 2018). However, previous studies have focused on astrocytic S100A10 in pathologies such as Alzheimer’s disease (King et al., 2020), ischemic stroke (Wang and Li, 2023), and other neurological disorders (Fei et al., 2022; Huang et al., 2024). For instance, cerebral ischemia activates A1/A2 astrocytes (Hernández et al., 2021). S100A10-positive A2 astrocytes augmented in the ischemic penumbra upregulate neurotrophic factors and vascular growth factors, presumably promoting neuronal survival, aiding axon regeneration, and promoting blood–brain barrier repair (Liddelow and Barres, 2017; Walker et al., 2020; Wang and Li, 2023). These data suggest that S100A10 or astrocyte S100A10 has a strong neuroprotective effect in AIS. However, until now, the specific role of neuronal S100A10 in stroke was poorly understood. Our present study extended the scope of known S100A10 roles. We showed that S100A10 was elevated and predominantly colocalized with neurons in the brain tissue of patients with AIS and MCAO/R rats, and was increased in the serum of patients with AIS and OGD/R neurons. S100A10 knockdown exacerbated PANoptosis, increased infarct volume, and worsened neurological deficits after ischemia. These results suggest that neuronal S100A10 provides important protection against cerebral ischemic injury. Notably, this study is the first to report elevated S100A10 levels in both brain tissue and serum of patients with AIS, highlighting its potential as a diagnostic biomarker or therapeutic target. However, conflicting evidence suggests S100A10 expression may follow a biphasic pattern—initially decreased then upregulated—after ischemic insult (Wang and Li, 2023). Our analysis focused on a single timepoint (3 days after ischemia), and did not determine whether the expression of S100A10 is upregulated or downregulated in the hyperacute and late stages of ischemia. The spatiotemporal expression of S100A10 should be dynamically detected in subsequent studies.

The activated Shh signaling pathway exhibits neuroprotective and repairing effects in a variety of pathological conditions, including cerebral ischemia, trauma, and inflammation. For example, pharmacological activation of Shh via resveratrol or smoothened agonist suppresses neuronal apoptosis, attenuates microglia/astrocytic activation, and promotes neurogenesis, synaptogenesis, and angiogenesis, ultimately improving functional recovery post-ischemia (Jin et al., 2017; Yu et al., 2017, 2021; Yang et al., 2021). Additionally, cerebral ischemia upregulated Shh expression, and this elevation in Shh expression showed a positive correlation with the development of fibrotic scar tissue. The modulation of Shh in BV2 cells, either through knockdown or activation, influenced fibroblast activation (Yang et al., 2024). Furthermore, Shh secreted by M2 macrophages promoted fibrogenesis in the initial phases following cerebral ischemia in rats (Huang et al., 2023). Here, our results showed that exogenous rShh downregulated PANoptosis-related protein expression, increased neuronal survival, and improved the outcome after OGD/R or MCAO/R injury, which indicates a function of Shh signaling that has not been previously reported.

Wang et al. (2021) reported that Shh controlled extracellular glutamate levels and affected ischemic brain damage in rodents by inhibiting GLT-1 membrane expression by PKCα phosphorylation of serine-562 on GLT-1. Activation or knockdown of Shh altered the expressions of TGFβ1 and PDGFA and controlled fibroblast activation (Yang et al., 2024). Moreover, Shh increased oxidative phosphorylation in macrophages, enhanced macrophage efferocytosis, and induced M2 polarization, finally promoting the progression of cutaneous scar formation (Zhang et al., 2024a). Here, we observed that rShh reversed the effects of S100A10 knockdown on PANoptosis and outcomes after MCAO/R injury. These findings indicate that Shh functions in several ways.

This study has some limitations that should be noted. We chose the MCAO/R model, which provides a classic research framework for ischemia–reperfusion injury, but future work should integrate the ET-1 model (simulating cortical small infarctions) and the light thrombus model (evaluating thrombus clearance interventions) for a comprehensive understanding. In particular, it is necessary to verify whether the neuroprotective effect of S100A10 is still significant in nonreperfusion injury scenarios (such as the ET-1 model), which will provide a basis for the precise treatment of different stroke subtypes in clinical practice.

In conclusion, our findings demonstrate for the first time that Shh may partially control the effects of S100A10 on PANoptosis and outcomes after cerebral ischemia. These insights provide a novel viewpoint on the regulatory dynamics of PANoptosis activation after stroke and present potential therapeutic avenues for enhancing neurological recuperation in patients with ischemic stroke. Nonetheless, the exact molecular pathway by which Shh controls S100A10 to affect PANoptosis activation, the role and mechanism of S100A10 in the ultra-early and middle-to-late stages of ischemic brain injury, and the transcription and posttranslational modification of key genes and proteins of PANoptosis need to be further studied.

## Data Availability

*The data used to support the findings of this study are included in the paper.*
